# Antimicrobial Activity, Biocompatibility and Anti-inflammatory Properties of Cetylpyridinium Chloride-based Mouthwash Containing Sodium Fluoride and Xylitol: An In Vitro Study

**DOI:** 10.3290/j.ohpd.b871071

**Published:** 2020-12-14

**Authors:** Geneviève LeBel, Katy Vaillancourt, Marie-Pierre Morin, Daniel Grenier

**Affiliations:** a Laboratory Technician, Oral Ecology Research Group (GREB), Faculty of Dentistry, Université Laval, Quebec City, Quebec, Canada. Performed the experiments, read and approved final manuscript.; b Dentist, Oral Ecology Research Group (GREB), Faculty of Dentistry, Université Laval, Quebec City, Quebec, Canada. Performed the experiments, read and approved final manuscript.; c Professor, Oral Ecology Research Group (GREB), Faculty of Dentistry, Université Laval, Quebec City, Quebec, Canada. Participated in experimental design, wrote manuscript, read and approved final manuscript.

**Keywords:** anti-inflammatory, antimicrobial, biofilm, cetylpyridinium chloride, mouthwash, xylitol

## Abstract

**Purpose:**

The use of a mouthwash as an adjunct to mechanical plaque removal may be useful to improve oral hygiene. In this study, cetylpyridinium chloride (CPC)-based mouthwashes containing sodium fluoride and xylitol (X-PUR Opti-Rinse 0.05% NaF and X-PUR Opti-Rinse 0.2% NaF) were evaluated for their antimicrobial activity against important oral pathogens associated with dental caries, periodontal disease, and candidiasis. Moreover, their biocompatibility and anti-inflammatory properties were assessed.

**Materials and Methods:**

Antimicrobial activity was determined using a disk-diffusion assay, a microplate dilution assay, and the European standard protocols for antiseptics. Microbicidal properties were assessed against both planktonic and biofilm cultures. An oral epithelial cell model was used to evaluate the biocompatibility of mouthwashes and their ability to attenuate cytokine secretion.

**Results:**

Using three different antimicrobial assays, the CPC-based mouthwashes were found to be highly active against the tested microorganisms. More specifically, the mouthwashes met the European Standard NF EN 1040 and NF EN 1275 defined as a log10 reduction ≥ 5 (≥ 99.999% killing) for bacteria and log10 reduction ≥ 4 (≥ 99.99% killing) for fungi, respectively. The CPC-based mouthwashes were also bactericidal against biofilms of *S. mutans, S. sobrinus,* and *P. gingivalis*. Using an oral epithelial cell model, the CPC-based mouthwashes were found to be less cytotoxic than a chlorhexidine-containing mouthwash used as control. Lastly, the CPC-based mouthwashes decreased the secretion of interleukin-6 and interleukin-8 by lipopolysaccharide-stimulated oral epithelial cells.

**Conclusion:**

The CPC-based mouthwashes supplemented with sodium fluoride (0.05% or 0.2%) and xylitol (10%) were highly active against important oral pathogens. Moreover, using an oral epithelial cell model, these mouthwashes were found to be biocompatible and to exhibit anti-inflammatory activity.

Dental plaque, also known as dental biofilm, is the primary etiological agent of oral infections, including dental caries and periodontal diseases. Appropriate oral hygiene is based on mechanical plaque removal through regular brushing and flossing. However, effective removal of dental plaque is not always possible, especially in handicapped or older individuals who may lack dexterity or motivation. To overcome those shortcomings, the use of a mouthwash as an adjunct to oral hygiene practices may be appropriate to achieve better control of dental plaque.^33^

Dental bioaerosols, which represent an occupational hazard in dental practice, are produced during dental procedures, especially when an ultrasonic scaler or high-speed drill is used.^16^,^38^ The bioaerosols contain mainly viruses and bacteria originating from blood, saliva, dental plaque and dental-unit waterlines, and may be involved in airborne-transmitted infections within the dental office.^16^,^38^ A number of studies have shown that pretreatment oral rinsing with an antiseptic mouthwash can significantly reduce the levels of bioaerosols generated during dental operative procedures.^9^,^26^ Consequently, this ever-more prevalent practice may decrease the risk of cross contamination in dental environments.

Oral rinsing was first reported in Chinese medicine in 2700 B.C. as a folk remedy.^35^ Today, active ingredients in mouthwashes are often cationic agents such as chlorhexidine (bisbiguanide) and cetylpyridinium chloride (CPC) (quaternary ammonium compound).^33^ While chlorhexidine has been extensively studied as chemical agent for plaque control,^5^,^25^,^28^ it is associated with several undesirable side-effects, including tooth staining, taste alterations, calculus formation, and oral mucosa desquamation/irritation.^1^,^37^ CPC, which exhibits a broad spectrum of antimicrobial activity, represents an interesting alternative to chlorhexidine as an antiseptic agent. Clinical studies showed that when incorporated into oral hygiene products, CPC significantly reduces dental plaque and gingivitis.^15^,^18^,^36^ Moreover, Rioboo et al^29^ reported that daily use of CPC-containing mouthwashes reduces oral malodour. Given the adverse effects of chlorhexidine, it has been proposed that this molecule may be more appropriate for acute/short-time use, while CPC may be more indicated for long-term maintenance treatment.^33^ This is further supported by the in vitro study of Kulik et al,^22^ who provided evidence that some bacterial species may develop resistance following prolonged exposure to chlorhexidine.

Although a large variety of mouthwashes have already been commercialized, companies are always looking for new formulations that can offer benefits over the others. With the intent to increase their therapeutic benefits, two new CPC-based mouthwashes supplemented with sodium fluoride (0.05% or 0.2%) and xylitol (10%) have recently been been developed and commercialized. In this study, these mouthwash formulations were evaluated for their antimicrobial activity against planktonic and biofilm cultures of important oral pathogens associated with dental caries (*Streptococcus mutans, Streptococcus sobrinus*), periodontal diseases (*Porphyromonas gingivalis*), and candidiasis (*Candida albicans*). Their biocompatibility and anti-inflammatory properties were investigated using an oral epithelial cell in vitro model.

## MATERIALS AND METHODS

### Mouthwashes

The mouthwashes tested in this study were X-PUR Opti-Rinse 0.05% sodium fluoride (NaF) and X-PUR Opti-Rinse 0.2% NaF (both from Oral Science; Longueuil, QC, Canada). Both contained 0.05% CPC and 10% xylitol. PerioGard (Colgate Oral Pharmaceuticals; Toronto, ON, Canada), which contains 0.12% chlorhexidine gluconate and 11.6% alcohol, was used as a positive antiseptic mouthwash control.

### Microorganisms and Growth Conditions

*S. mutans *ATCC 25175 and *S. sobrinus* ATCC 33478 were cultivated in Todd-Hewitt broth (THB, BBL Microbiology Systems; Mississauga, ON, Canada), while *C. albicans* ATCC 28366 was grown in Yeast Nitrogen Base (YNB) broth (BBL Microbiology Systems) containing 0.5% glucose; incubation was carried out at 37°C under aerobic conditions. *P. gingivalis* ATCC 33277 was grown in THB supplemented with hemin (10 µg/ml) and vitamin K (1 µg/ml) and incubated at 37°C in an anaerobic chamber (80% N_2_/10% H_2_/10% CO_2_).

### Disk-diffusion Assay for Determination of Antimicrobial Activity

The antimicrobial activity of mouthwashes was first assessed by a disk-diffusion assay. Briefly, disks (6 mm diameter) of cellulose paper (BBL Microbiology Systems) were moistened with 25 µl of mouthwashes and allowed to dry at room temperature. Overnight cultures of microorganisms under investigation were spread (200 µl) on the surface of solid culture media. The disks were then applied onto the surface of plates, which were immediately incubated under the appropriate culture conditions. Following bacterial growth (up to 4 days), the radius of the inhibition zone was measured from the edge of the paper disk to the margin of the inhibition area. All experiments were performed in triplicate and the means ± SD of the inhibitory zones were calculated.

### Determination of Minimum Inhibitory and Minimum Microbicidal Concentrations

A microplate dilution assay was used to determine the minimum inhibitory concentration (MIC) and minimum microbicidal concentration (MMC) of the mouthwashes. To determine MIC, 24-h microbial cultures were diluted in fresh culture medium to obtain an optical density of 0.1 at 660 nm (OD660). Equal volumes (100 µl) of the suspensions and two-fold serial dilutions of mouthwashes (from 6.25%; v/v) in culture medium were added to the wells of a 96-well microplate. Wells with no mouthwash were used as controls (100% growth). After 24-h incubation, microbial growth was monitored by recording the OD660 using a Synergy 2 microplate reader (BioTek Instruments; Winooski, VT, USA). The MIC was the lowest concentration of mouthwashes that completely inhibited microbial growth. To determine the MMC values, 5-μl aliquots from the wells with no visible growth were spread on appropriate culture agar plates. After an incubation of 5 days at 37°C, the MMC was determined as the lowest concentration at which no colony formation occurred. Assays were performed in triplicate to ensure reproducibility and a representative set of data is presented.

### Determination of Antimicrobial Activity According to European Standard NF EN 1040 and NF EN 1275

Microbicidal activity of mouthwashes was determined according to the European Standard NF EN 10402 for *S. mutans, S. sobrinus*, and *P. gingivalis*, and the European Standard NF EN 12758 for *C. albicans*. Briefly, an overnight culture (3 ml) of bacteria was vigorously mixed in the presence of sterile 0.3- to 0.5-mm glass beads (0.2 g; Sigma-Aldrich Canada; Oakville, ON, Canada) for 1 min to break down chains and aggregates of microorganisms. Then, bacteria (final concentration of 10^8^ cfu/ml) or *C. albicans* (final concentration of 10^7^cfu/ml) were added to mouthwashes (final concentration of 80%) in the presence of 0.3% bovine serum albumin as interfering agent. Bacteria added to 50 mM phosphate-buffered saline (PBS; pH 7.2) were used as control (100% viable bacteria). Following a 5-min exposure at 20°C, the antiseptic property of mouthwashes was stopped by adding a neutralizing solution (Dey-Engly neutralizing broth, Sigma-Aldrich Canada. Tenfold serial dilutions were then prepared in Tryptone (BBL Microbiology Systems) sodium chloride solution (0.1% Tryptone + 0.85% sodium chloride; pH 7.0) and viable cell counts were determined by plating in triplicate on the appropriate culture agar plates. All plates were incubated under their appropriate culture conditions for 2 to 4 days. Following growth, colony-forming units (CFU) were calculated. According to the European Standards NF EN 1040 and NF EN 1275, a microbicidal antiseptic is defined for a log10 reduction ≥ 5 (≥ 99.999% killing) for bacteria and ≥ 4 (≥ 99.99% killing) for fungi, respectively. The effect of a 1-min exposure was also tested to take into consideration a more representative contact time of a mouthwash. The means ± SD were calculated. Assays were performed in triplicate and a representative set of data is presented.

### Determination of Biofilm Killing

The ability of mouthwashes to kill *S. mutans, S. sobrinus, P. gingivalis*, and *C. albicans* biofilms was investigated. Briefly, 24-h biofilms were pre-formed by growing microorganisms in wells of a 96-well microplate. Spent media and planktonic microorganisms were removed by aspiration using a 26-g needle, and biofilms were then treated for 5 min with mouthwashes or PBS (100 µl). Following these treatments, the biofilms were washed once in PBS. Biofilms from three wells were then detached by scraping, pooled, and the microorganisms suspended in PBS by vortexing (1 min) in the presence of sterile 0.3- to 0.5-mm glass beads (0.01 gram /300 µl; Sigma-Aldrich Canada). Tenfold serial dilutions were immediately prepared in Tryptone sodium chloride solution and viable cell counts were determined by plating in triplicate on the appropriate culture agar plates. Following growth, colony-forming units (CFU) were calculated. In the case of *P. gingivalis*, the dilutions (5 µl) were inoculated into liquid culture medium (10 ml). All plates and tubes were incubated under their appropriate culture conditions for 2 to 4 days. A series of three biofilms treated as above was stained with 0.01% crystal violet as previously described^4^ to determine whether the treatment with mouthwashes caused desorption of the biofilm. The means ± SD were calculated.

### Biocompatibility of Mouthwashes in an Oral Epithelial Cell Model

The human oral epithelial cell line GMSM-K^13^ (Department of Diagnostic Sciences and General Dentistry, University of North Carolina, Chapel Hill, NC, USA) was cultured in Dulbecco’s modified Eagle’s medium (DMEM) supplemented with 10% heat-inactivated fetal bovine serum (FBS) and 100 µg/ml of penicillin G/streptomycin at 37ºC in a 5% CO_2_ atmosphere. The cells were harvested by gentle trypsinization (0.05% trypsin-EDTA), washed once in DMEM-FBS, and suspended at a density of 4 x 10^5^ cells/ml in DMEM supplemented with 1% heat-inactivated FBS. The cells were seeded in 96-well plates (100 µl/well) and were cultured overnight at 37°C in a 5% CO_2_ atmosphere to allow cell adhesion prior to the treatment. Epithelial cells were treated for 1 and 2 min with mouthwashes (diluted 1:2, 1:4, 1:8, and 1:16 in culture medium) and wells were immediately washed with DMEM prior to assessing cell viability using an MTT (3-[4, 5-diethylthiazol-2-yl]-2,5-diphenyltetrazolium bromide) assay performed according to the manufacturer’s protocol (Roche Diagnostics; Mannheim, Germany). Assays were carried out in triplicate, and the means ± SD were calculated.

### Anti-inflammatory Activity of Mouthwashes in an Oral Epithelial Cell Model

GMSM-K epithelial cells were cultivated as described above and pre-treated with mouthwashes (1:256 dilution) for 2 h. The culture medium containing the mouthwash was removed prior to adding fresh media containing lipopolysaccharide (LPS; 1 µg/ml) isolated from *Aggregatibacter actinomycetemcomitans* according to the protocol of Darveau and Hancock^7^ for 24 h at 37°C in a 5% CO_2_ atmosphere. The cell-free supernatants were collected and subsequently stored at -20°C until use. Commercial enzyme-linked immunosorbent assays (ELISA) kits (R&D Systems; Minneapolis, MN, USA) were used to quantify IL-6 and IL-8 concentrations in the supernatant of treated cells according to the manufacturer’s protocols. All experiments were carried out in triplicate, and the means ± SD deviations were calculated.

### Statistical Analysis

Statistical analyses were performed using a one-way ANOVA with a post-hoc Bonferroni multiple comparison test (GraphPad Software; La Jolla, CA, USA). All results were considered statistically significant at p < 0.01.

## RESULTS

Three experimental procedures were used to assess the in vitro antimicrobial activity of the two commercial CPC-based mouthwashes against cariogenic bacteria (*S. mutans, S. sobrinus*), a periodontal pathogen (*P. gingivalis*), and a pathogenic fungus (*C. albicans*). Table 1 reports the growth inhibition zones obtained using a disk-diffusion method. X-PUR Opti-Rinse 0.2% NaF produced slightly larger zones of growth inhibition against *S. mutans* (5.5 ± 2.3 mm vs 4.5 ± 0.7 mm), *S. sobrinus* (4.5 ± 1.9 mm vs 4.0 ± 1.2 mm), and *P. gingivalis* (5.8 ± 1.4 mm vs 5.4 ± 2.0 mm) in comparison with X-PUR Opti-Rinse 0.05% NaF. Smaller growth inhibition zones, 0.7 ± 0.3 mm and 0.4 ± 0.2 mm, were obtained against *C. albicans* for X-PUR Opti-Rinse 0.05% NaF and X-PUR Opti-Rinse 0.2% NaF, respectively. The chlorhexidine-based mouthwash used as positive control produced larger inhibitory zones, particularly against *P. gingivalis* (14.3 ± 2.1 mm) and *C. albicans* (2.5 ± 0.2 mm).

**Table 1:** Minimum inhibitory concentrations (MIC), minimum microbicidal concentrations (MMC), and growth inhibition zones (means ± SD) of mouthwashes against oral microbial pathogens

Micro-organism	Antimicrobial activity^1^								
	X-PUR Opti-Rinse + 0.05% NaF			X-PUR Opti-Rinse + 0.2% NaF			Chlorhexidine-based mouthwash		
MIC	MMC	Inhibitory zone (mm)	MIC	MMC	Inhibitory zone (mm)	MIC	MMC	Inhibitory zone (mm)
*S. mutans*	0.39%	0.39%	4.5 ± 0.72	0.78%	1.56%	5.5 ± 2.3	0.195%	0.195%	6.8 ± 0.3
*S. sobrinus*	0.39%	0.39%	4.0 ± 1.2	0.78%	0.78%	4.5 ± 1.9	0.195%	0.39%	7.1 ± 1.3
*P. gingivalis*	0.39%	0.39%	5.4 ± 2.0	1.56%	1.56%	5.8 ± 1.4	0.39%	0.39%	14.3 ± 2.1
*C. albicans*	0.78%			1.56%			0.39%	0.78%	2.5 ± 0

^1^ MIC and MMC were determined using a microplate dilution assay. Growth inhibition zones were determined on culture plates using a disk-diffusion assay.

Thereafter, the MIC and MMC values of mouthwashes were determined using a microplate dilution assay (Table 1). In general, the values were slightly lower with X-PUR Opti-Rinse 0.05% NaF than with X-PUR Opti-Rinse 0.2% NaF. For all three bacterial species tested, the MIC and MMC values of X-PUR Opti-Rinse 0.05% NaF were 0.39%, while the values were 0.78% for *C. albicans*. With regard to X-PUR Opti-Rinse 0.2% NaF, the MIC and MMC values ranged from 0.78% to 1.56% for all microorganisms tested. The MIC and MMC values of the chlorhexidine-based mouthwash ranged from 0.195% to 0.78%.

**Fig 1:**
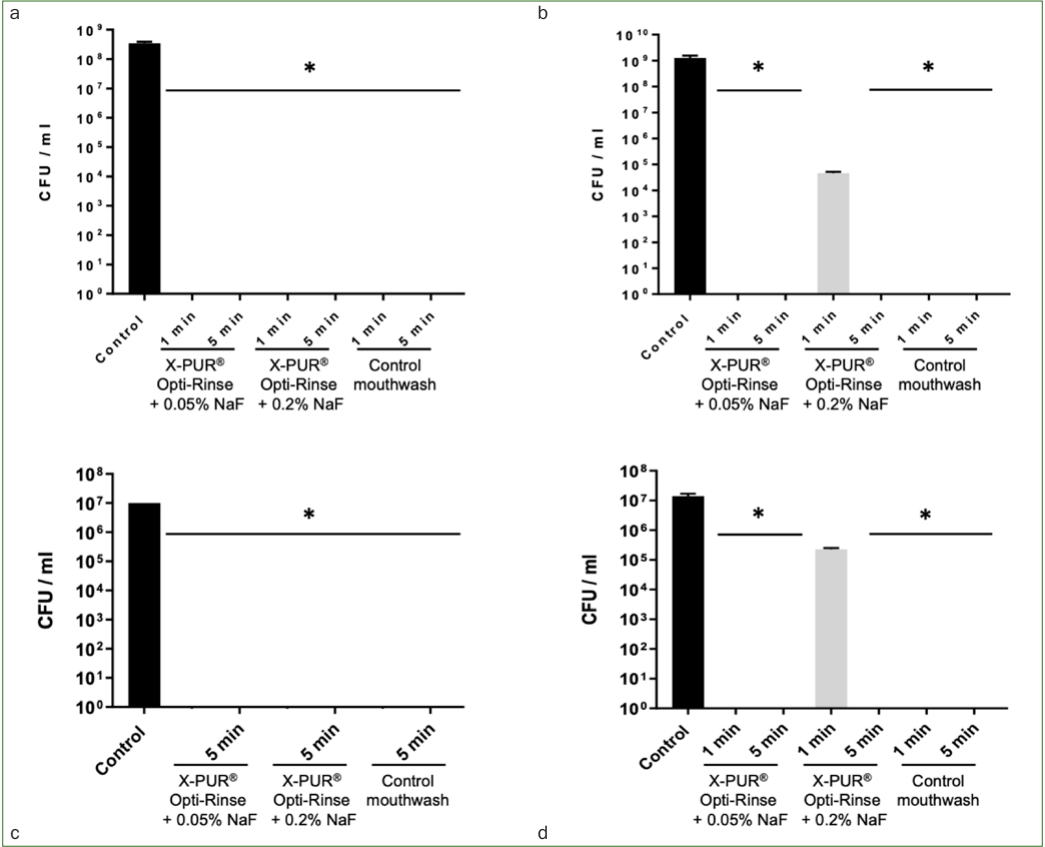
Log10 reduction (CFU/ml) of *S. mutans* (panel a), *S. sobrinus* (panel b), *P. gingivalis* (panel c), and *C. albicans* (panel d) following a time contact of 1 min and 5 min in the presence of mouthwashes. *The mouthwash meets the European Standard NF EN 1040 or NF EN 1275 defined as a log10 reduction ≥ 5 (≥ 99.999% killing) for bacteria and log10 reduction ≥ 4 (≥ 99.99% killing) for fungi.

Lastly, the antimicrobial activity of the mouthwashes was assessed according to the European Standard NF EN 1040 (for bacteria) and NF EN 1275 (for fungi) protocols. Preliminary analyses showed that the neutralizing solution (Dey-Engly neutralizing broth) used to stop the microbicidal effect of the mouthwashes had no direct effect on the viability of the microorganisms tested (data not shown). Following a contact time of 5 min (as required for the European standards), the logarithmic reductions in CFU observed for each microorganism are presented in Fig 1. The two CPC-based mouthwashes under investigation as well as the chlorhexidine-containing mouthwash control allowed > 5 log reduction of *S. mutans, S. sobrinus*, and *P. gingivalis*, and > 4 log reduction of *C. albicans* following a contact time of 5 min. This indicates that the two mouthwashes examined met the European Standards against the tested microorganisms. We also tested whether the European Standards can be satisfied following an exposition of 1 min, a contact time that may be more realistic for a mouthwash. With the exception of X-PUR Opti-Rinse 0.2% NaF against *C. albicans*, the standards were met.

Mouthwashes were further investigated for their ability to kill biofilm-grown microorganisms (Table 2). The X-PUR Opti-Rinse 0.05% NaF and X-PUR Opti-Rinse 0.2% NaF decreased the biofilm viability of *S. mutans* and *S. sobrinus* in the range of 87.5% to 96.7%. These effects were more pronounced than those obtained with the chlorhexidine-based mouthwash control. Both the CPC- and chlorhexidine-based mouthwashes killed most (≥ 99%) of the *P. gingivalis* biofilm. Regarding the *C. albicans* biofilm, while the X-PUR Opti-Rinse (0.05% and 0.2% NaF) did not decrease its viability, the chlorhexidine-based mouthwash decreased the viability by 66.8%. The X-PUR Opti-Rinse (0.05% and 0.2% NaF) did not cause desorption of the *S. mutans, S. sobrinus*, and *P. gingivalis*, while some desorption was observed for the *C. albicans* biofilms (Table 2).

**Table 2:** Effects of mouthwashes on killing and desorption of oral microbial pathogen biofilms (means ± SD)

Microorganism	Mouthwash					
	X-PUR Opti-Rinse + 0.05% NaF		X-PUR Opti-Rinse + 0.2% NaF		Chlorhexidine-based mouthwash	
	Killing (%)	Desorption (%)	Killing (%)	Desorption (%)	Killing (%)	Desorption (%)
*S. mutans*	96.7 ± 0.4%	0	96.4 ± 0.2%	0	68.0 ± 1.4%	0
*S. sobrinus*	87.5 ± 0.5%	0	87.5 ± 1.7%	0	83.7 ± 0.3%	0
*P. gingivalis*	99.9% ± 0%	0	99% ± 0%	0	99.9% ± 0%	0
*C. albicans*	No effect	16.2 ± 7.5	No effect	27.4 ± 4.4	70.8 ± 0.4%	6.5 ± 3.5

**Fig 2:**
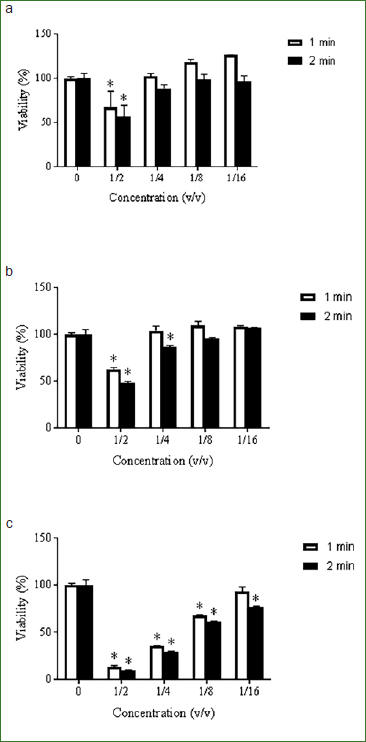
Effects of mouthwashes on viability of oral epithelial cells following a contact time of 1 and 2 min. Cell viability was assessed using an MTT colorimetric assay. Results are expressed as the means ± SD of triplicate assays. *Statistically significant decrease (p < 0.01) compared to control untreated epithelial cells.

The in vitro cytotoxicity of the mouthwashes under investigation was then evaluated using an oral epithelial cell model. As reported in Fig 2, following a contact time of 1 and 2 min, the X-PUR Opti-Rinse (0.05% and 0.2% NaF) was found to be less cytotoxic than the chlorhexidine-based mouthwash control. More specifically, following a 1-min exposure at a 1/4 dilution, the X-PUR Opti-Rinse (0.05% and 0.2% NaF) did not cause any significant reduction of epithelial cell viability, while the chlorhexidine-based mouthwash reduced viability by 64.4%.

**Fig 3:**
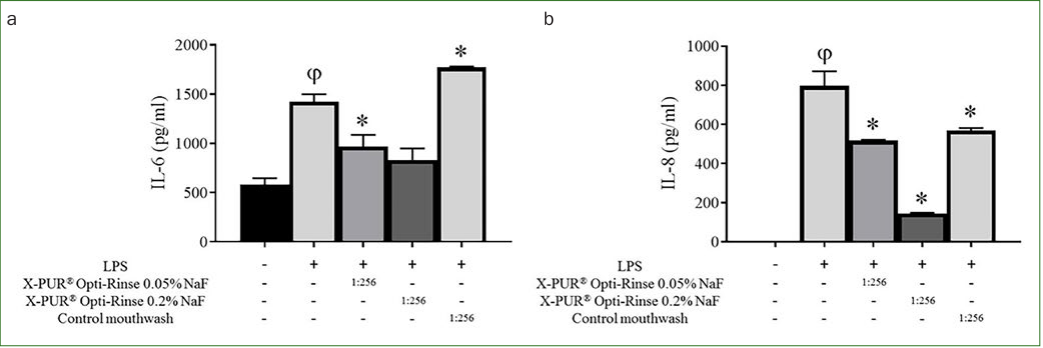
Effects of mouthwashes on IL-6 (panel A) and IL-8 (panel B) secretion by oral epithelial cells stimulated with LPS. Cytokine levels in culture medium supernatants were determined by ELISA. Results are expressed as the means ± SD of triplicate assays. ϕStatistically significant increase (p < 0.01) compared to control untreated cells. *Statistically significant decrease (p < 0.01) compared to LPS-stimulated epithelial cells.

To assess the anti-inflammatory properties of the mouthwashes, their ability to inhibit IL-6 and IL-8 secretion by oral epithelial cells challenged with LPS was tested. Preliminary assays showed that to avoid any cytotoxic effects related to the long exposure time (2 h) of epithelial cells to mouthwashes, a 1:256 dilution had to be used (data not shown). LPS stimulation of epithelial cells significantly increased the secretion of both IL-6 and IL-8 (Fig 3). The X-PUR Opti-Rinse 0.05% NaF and X-PUR Opti-Rinse 0.2% NaF decreased IL-6 secretion by 32.1% and 41.6%, respectively, while the chlorhexidine-based mouthwash did not reduce the secretion of IL-6 by LPS-stimulated oral epithelial cells. With regard to IL-8 secretion, the X-PUR Opti-Rinse 0.05% NaF and X-PUR Opti-Rinse 0.2% NaF caused a reduction of 35.1% and 81.9%, respectively, while the chlorhexidine-based mouthwash reduced IL-8 secretion by 28.8%. 

## DISCUSSION

Poor oral hygiene that results in dental plaque accumulation has negative impacts on oral health, since it can lead to caries and periodontal diseases. Moreover, poor oral hygiene causes dysbiosis of the oral microbiome, thus contributing to chronic inflammation that may result in a number of systemic pathological conditions, including cardiovascular disease, rheumatoid arthritis, and diabetes mellitus.^23^ Daily preventive oral care, including proper brushing and flossing, can help prevent these disorders. Nowadays, many people use oral mouthwashes in their home-care regimen for the chemical control of dental biofilm as an adjunct to the mechanical procedures.^3^,^33^ The use of an oral antiseptic as a pre-procedural mouthrinse has also been proposed as a preventive measure to reduce bioaerosol formation during dental procedures, thus decreasing the risk for infectious agent transmission in dental practice.^9^,^26^

Chlorhexidine is considered the gold-standard oral antiseptic due to its high clinical and microbiological efficacy.^5^,^25^,^28^ However, a number of undesired side-effects such as tooth staining, taste alteration, calculus formation, and oral mucosa desquamation/irritation have been associated with its use.^1^,^37^CPC is an amphilic quaternary compound with a broad antimicrobial activity spectrum that is considered as an alternative to chlorhexidine. The antimicrobial action of CPC relates to its ability to non-specifically bind to microbial cell surfaces, inducing disruption of the cell membrane and thus cell death.^12^ The clinical benefits of CPC-based mouthwashes, with regard to reduction of dental plaque and gingivitis, have been previously reported.^15^,^17^,^18^,^30^ Although some studies have evaluated the antimicrobial property of CPC-containing mouthwashes against oral pathogens^24^,^31^ very little is known regarding their effects on oral mucosal cells. In this study, CPC-based mouthwashes supplemented with sodium fluoride and xylitol (X-PUR Opti-Rinse 0.05% NaF and X-PUR Opti-Rinse 0.2% NaF) were evaluated for their antimicrobial activity against planktonic cells and biofilms of important oral pathogens. Their biocompatibility and anti-inflammatory properties were also investigated using an oral epithelial cell in vitro model. A chlorhexidine-based mouthwash was included for comparison.

Different protocols can be used to investigate the antimicrobial activity of mouthwashes. In this study, three procedures (disk-diffusion method, broth microdilution assay, European standard protocol) confirmed the high antimicrobial activity of the CPC-based mouthwashes against bacteria (*S. mutans, S. sobrinus, P. gingivalis*) and a fungus (*C. albicans*). More specifically, the in vitro microbicidal assays conducted in accordance with the protocol of European standards for antiseptics showed that the mouthwashes meet the European Standard NF EN 1040 defined as a log10 reduction ≥ 5 (≥ 99.999% killing) for bacteria and the NF EN 1275 defined as a log10 reduction ≥ 4 (≥ 99.99% killing) for fungi following a 5-min contact time. Interestingly, the standards were met even after a short exposure of 1 min, which is more representative of contact time when using a mouthwash. This protocol includes the use of an interfering agent and a neutralizing solution. The purpose of using an interfering agent (0.3% bovine serum albumin) is to mimic an organic load that may interfere with the efficiency of an antiseptic solution. The importance of using a neutralizing solution is to prevent any residual antimicrobial effect of the active compounds following exposure to microorganisms and during sample processing. Consequently, this allows accurate determination of the antimicrobial effect for a specific exposure time. Kampf et al^21^ showed that evaluating the antimicrobial properties of compounds without using neutralizing agents may overestimate product efficacy. Using the broth microdilution method, the CPC-based mouthwashes showed MMC values in the range of 0.39% to 1.56% against all oral pathogens tested. This suggests that even diluted (as it can be with saliva), the mouthwash may be still exhibit antimicrobial activity.

In addition to conduct antimicrobial susceptibility assays using planktonic microorganisms, we also tested the CPC-based mouthwashes for their antimicrobial effects on biofilm cultures, which are known to be markedly more resistant than their planktonic counterparts. Although, the mouthwashes examined here showed weaker microbicidal effects against biofilms than against planktonic microorganisms, a marked reduction in biofilm viability was observed for *S. mutans, S. sobrinus*, and *P. gingivalis*.

In addition to having a broad antimicrobial spectrum, the ideal antiseptic mouthwash should not be toxic for mucosal cells. Using an oral epithelial cell model, this study demonstrated that the CPC-based mouthwashes are significantly less cytotoxic that the chlorhexidine-containing control mouthwash for contact times of 1 and 2 min. The cytotoxicity of chlorhexidine has been previously demonstrated on human gingival fibroblasts^20^ and human periodontal ligament cells.^6^

The oral epithelium represents an effective physical barrier against oral pathogens.^14^ However, in the presence of an inflammatory condition, as observed in patients affected by periodontal disease, there may be a loss of the epithelial integrity, allowing oral pathogens to invade the underlying connective tissue. The ability of the CPC-based mouthwashes to attenuate the inflammatory response of oral epithelial cells was thus investigated. The present results showed that X-PUR Opti-Rinse 0.2% NaF and, to a lesser extent, X-PUR Opti-Rinse 0.05% NaF inhibit LPS-induced IL-6 and IL-8 secretion by oral epithelial cells. Recently, xylitol (≥ 0.5%) has been reported to be anti-inflammatory, in that it decreases the production of inflammatory mediators, including IL-1β and TNF-α, in a model of macrophages challenged by *P. gingivalis*.^27^ Therefore, part of the ability of the X-PUR Opti-Rinse formulations to decrease cytokine production may be associated with the presence of xylitol. This ability of the CPC-based mouthwashes to have anti-inflammatory action may have a beneficial impact, since it has been reported that IL-6 levels are higher in the diseased gingiva of patients with periodontitis than in the gingiva of periodontally healthy subjects.^32^ Moreover, increased levels of IL-8, a chemoattractant for polymorphonuclear leukocytes and macrophages, have been found in the gingival crevicular fluid of inflamed periodontal sites compared with healthy sites.^10^ Interestingly, periodontal therapy reduces immune cell numbers as well as the levels of IL-8 in gingival crevicular fluid.^11^

Mouthwashes are often complex mixtures that contain, in addition to the main antimicrobial agent, a number of other ingredients that may exert some additional beneficial effects with regard to oral health. The CPC-based mouthwashes under investigation contain sodium fluoride (0.05% or 0.2%) as well as xylitol (10%). Sodium fluoride may provide a preventive effect against caries by promoting remineralization of enamel,^34^ while xylitol is known to possess anti-cariogenic properties through its action on cariogenic bacteria.^19^

## CONCLUSIONS

The present study showed that X-PUR Opti-Rinse 0.05% NaF and X-PUR Opti-Rinse 0.2% NaF, containing CPC as antimicrobial agent, meet the European Standards against the oral pathogens tested. Moreover, using an oral epithelial cell model, these mouthwashes were found to be biocompatible and to possess anti-inflammatory activity. These data support the evaluation of their effects on dental plaque and gingivitis in future clinical studies. 
